# IOPA: I/O-aware parallelism adaption for parallel programs

**DOI:** 10.1371/journal.pone.0173038

**Published:** 2017-03-09

**Authors:** Tao Liu, Yi Liu, Chen Qian, Depei Qian

**Affiliations:** 1 Sino-German Joint Software Institute, Beihang University, Beijing, China; 2 State Key Laboratory of Mathematical Engineering and Advanced Computing, Wuxi, China; University of the West of England, UNITED KINGDOM

## Abstract

With the development of multi-/many-core processors, applications need to be written as parallel programs to improve execution efficiency. For data-intensive applications that use multiple threads to read/write files simultaneously, an I/O sub-system can easily become a bottleneck when too many of these types of threads exist; on the contrary, too few threads will cause insufficient resource utilization and hurt performance. Therefore, programmers must pay much attention to parallelism control to find the appropriate number of I/O threads for an application. This paper proposes a parallelism control mechanism named IOPA that can adjust the parallelism of applications to adapt to the I/O capability of a system and balance computing resources and I/O bandwidth. The programming interface of IOPA is also provided to programmers to simplify parallel programming. IOPA is evaluated using multiple applications with both solid state and hard disk drives. The results show that the parallel applications using IOPA can achieve higher efficiency than those with a fixed number of threads.

## 1 Introduction

In the era of big data, data intensive applications become common in both desktop and server systems, many of which need to access large amount of files including web pages, images, and videos. At the same time, as multi-/many-core processors become ubiquitous, these data-intensive applications need to be written as parallel programs to make efficient use of the available computing resources.

Due to the performance gap between CPU and I/O sub-system, I/O bandwidth can easily become system bottleneck, especially for parallel applications that use multiple threads to read/write files simultaneously. In this situation, multiple I/O threads may generate too many requests that overload the I/O sub-system, and the different requests may interfere with each other in prefetching, buffering and disk actions, causing further reductions in I/O throughput. On the contrary, if the number of I/O threads is too few, the I/O capability of the system cannot be fully utilized, and processor cores are wasted. Therefore, to make full use of I/O bandwidth while avoiding I/O bottlenecks, programmers must pay much attention to the I/O parallelism of a program, i.e., the number of threads that access files. Moreover, I/O parallelism control will be more complicated if considering the differences of I/O capabilities among different hardware platforms, e.g., high-end servers equipped with a powerful RAID card, desktops or low-end servers with common SATA disks.

To solve the parallelism control problem discussed above, this paper proposes IOPA, an I/O parallelism control mechanism that can adapt to the I/O capability of the system. The IOPA monitors the load of an I/O sub-system and correspondingly adjusts the number of I/O threads in applications. A programming interface is also provided to programmers to enable automated adjustment according to the current I/O throughput.

The rest of this paper is organized as follows. Section 2 discusses the motivation of this paper. Section 3 presents the I/O-aware parallelism adaption mechanism, parallelism adjustment algorithm and the IOPA programming interface. Section 4 details the system architecture and implementation. Evaluations with both solid state and hard disk drives are presented in Section 5 and Section 6 introduces related work. Finally, Section 7 provides conclusions.

## 2 Motivation

To exploit the relationship between the I/O parallelism and performance of the applications, we evaluate the overall performance of programs under different numbers of I/O threads. The programs used are micro-benchmarks that read specified files one by one of five kinds sizes: 10 KB, 50 KB, 100 KB, 500 KB and 1000 KB. Each of them has a total volume of 15 GB. The hardware used in the experiments is a dual-way x86 server equipped with two Intel Xeon 5650 processors (12 physical cores, Hyper-Threading, 24 logical cores in total) and 16 GB of memory. We respectively use a solid state (Samsung 850 Pro, 256 G) and a hard disk (TOSHIBA enterprise hard-disks, 10000 RPM, 32 MB cache, 300 GB) drive in the tests.

**Fig 1:**
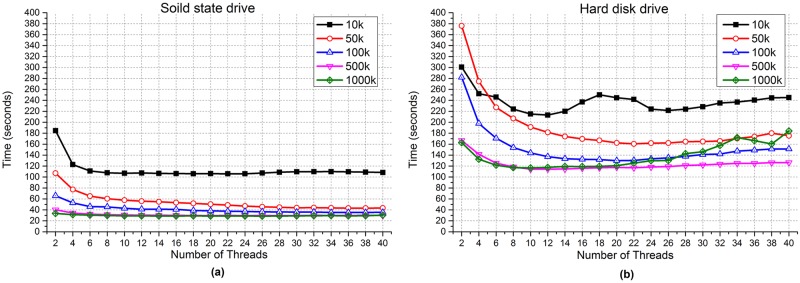
Performances with an SSD and an HDD while varying the numbers of threads. (a) SSD (solid state drive).(b) HDD (hard disk drive).

Fig 1 shows the evaluation results. As shown in the figure, there exist inflection points in the performance of the program, no matter the disk type or the file size. To the left of the inflection point, performance can benefit from increasing the number of threads, while to the right side of the inflection point, performance remains stable as the number of threads increases, or sometimes even becomes worse.

In the test, the number of threads corresponding to the inflection point can be considered as the appropriate number of threads for the application. However, this “appropriate number” depends on multiple factors, including the application logic, hardware resources and current load of the system. In other words, the exact inflection point is not only application-specific but also platform-dependent. Therefore, it is necessary to design a parallelism control mechanism that can adapt to the I/O capability of the system and adjust the number of I/O threads in parallel applications according to the load of the I/O sub-system, as well as mitigate the problem where excessive threads interfere with each other.

The reason why the results for SSD are more stable than for HDD is because that, the I/O behaviors of HDD are subject to its head, while SSD does not have a head component but is equipped with flash memory. The operating mechanisms of SSD and HDD are also different. Moreover, the temperature of HDD’s head increases while a program is reading or writing data, which may also cause fluctuations in I/O throughput.

## 3 I/O-aware parallelism adaption mechanism

### 3.1 Overview

The main objectives of IOPA are to adjust the number of threads in applications to an appropriate value that matches the I/O capability of the system and to simplify the programming. As discussed in the previous sections, the “appropriate number of threads” is not only application-specific but also platform-dependent; moreover, it is related to the current load of the system. With such heuristics, IOPA employs a dynamic approach that can adjust the number of threads periodically, based on the load of the system.

The principles of IOPA can be described as follows:

1. IOPA monitors the I/O throughput of the file system and periodically transmits the acquired statistics to IOPA’s runtime system.
2. IOPA uses a parallelism adjustment algorithm to calculate the appropriate number of threads for an application according to the I/O throughput, the available computing resources, and the current number of threads and set the number of threads when necessary.
3. A simple programming interface is provided to application programmers to use IOPA.

### 3.2 Parallelism adjustment algorithm

As the essence of IOPA, the parallelism adjustment algorithm calculates the appropriate number of threads for the program periodically based on the I/O throughput of the system, the available computing resources and the current load of the program. When necessary, it adjusts the number of threads used by the program.

The algorithm supports two types of working modes: *fast mode* and *normal mode*. *Fast mode* is used to adjust the number of threads to reach “near-optimal” quickly; subsequently, the algorithm switches to *normal mode*.

The *fast mode* adjustment cycle is half the length of the *normal mode* adjustment cycle. If the I/O throughput increases obviously (e.g., double) after incrementing the number of threads, then the value should be doubled in the next adjustment cycle until a preset threshold is reached or the increase in I/O throughput becomes insignificant.

Subsequently, the algorithm switches to *normal mode*; the duration of adjustment cycle in this mode is longer. According to the changes in I/O throughput, the algorithm periodically increases, decreases or maintains the current number of threads. Considering that I/O throughput sometimes fluctuates due to burst read/write operations in applications, the algorithm compares the current I/O throughput with the average I/O throughput in the previous *N* cycles to mitigate excessive influence from such fluctuations.

The parallelism adjustment algorithm aims to reduce the adjustment time and improve the performance of the adjusted application. Meanwhile, in *normal mode*, frequent adjustments to the number of threads are inefficient and unnecessary. Therefore, the adjustment cycles for each mode are different.

**Fig 2:**
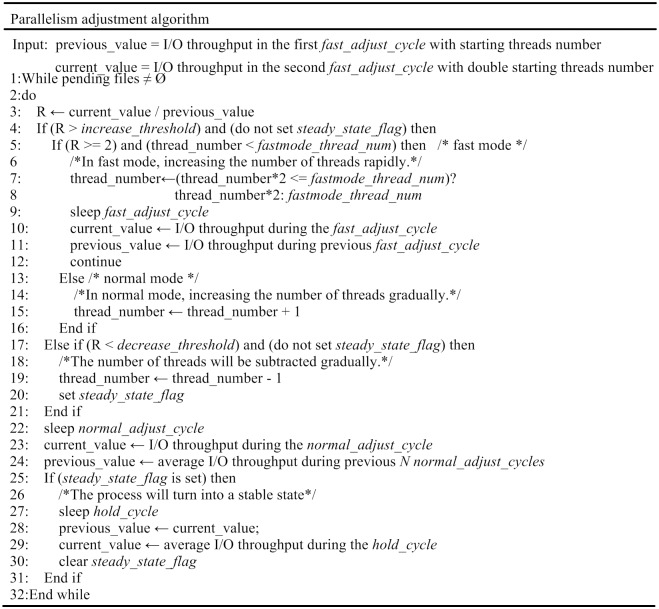
Parallelism adjustment algorithm of IOPA.

The pseudocode for the parallelism adjustment algorithm is shown in Fig 2, where *R* is the ratio of I/O throughput in the current adjust_cycle to that in the previous adjust_cycle; *fastmode_thread_num* is the maximum number of threads in *fast mode*; *fast_adjust_cycle* is the adjustment cycle duration in *fast mode*; *normal_adjust_cycle* is the adjustment cycle duration in *normal mode*, and *hold_cycle* is a cycle that maintains a stable state. To keep a parallel application stable, the algorithm uses two adjustment thresholds: *increase_threshold* and *decrease_threshold*. These thresholds exist to avoid frequent and unnecessary adjustments of the number of threads. The adjustments (increasing or decreasing the number of threads) occur only when the changes in I/O throughput exceed the preset thresholds.

In *fast mode*, if *R* exceeds the *increase_threshold*, the algorithm will increase the number of threads rapidly (lines 7~8 in Fig 2). In *normal mode*, if the increase in I/O throughput is smooth, the number of threads is incremented gradually (line 15 in Fig 2). When *R* falls below the *decrease_threshold*, the number of threads will be decreased (line 19 in Fig 2), and the steady state flag will be set (line 20 in Fig 2), which means that the process will switch to a stable state, and the current number of threads will be held constant for a *hold_cycle* (lines 27~30 in Fig 2). To avoid frequent adjustments or thrashing in the number of threads, the *hold_cycle* should be set longer than the *normal_adjust_cycle* (e.g., several times the *normal_adjust_cycle* in length).

$$Speedup=\frac{1}{r_{s}+\frac{r_{p}}{n}}$$ (1)

Formula (1) shows Amdahl’s law [1], where *r*_*s*_ + *r*_*p*_ = 1. Here, *r*_*s*_ and *r*_*p*_ represent the ratios of the sequential portion and parallel portion of one program, respectively, and *n* is the degree of parallelism. The speedup definitely depends on the value of *n*, which is a fixed value.

Moreover, the I/O throughput of entire system, the available computing resources and the current load of the program change constantly during the execution of a program. The optimal degree of parallelism, such as the optimal fixed number of processes/threads, is difficult to determine in an ever-changing execution environment. The parallelism adjustment algorithm can adjust the number of I/O threads based on the I/O throughput of the entire system, meaning that IOPA can guarantee that the degree of parallelism (*n*) will change over a reasonable range. This approach is both more flexible and more efficient for programs than is relying on a fixed number of threads.

### 3.3 Programming interface

The programming interface of IOPA consists of 4 API functions, as shown in Table 1. The kernel interface function is *IOPA_CreateThreads()*, which is similar to *pthread_create()* in the pthread API[2] or *CreateThread()* in the Windows API[3], and is used to tell IOPA the entry of the thread function in the upper layer application. After this interface function is invoked, IOPA will create multiple working threads that execute a user-defined thread function and adjust the number of threads adaptively. Subsequently, the application invokes *IOPA_WaitComplete()* to await termination of all working threads. The user-defined thread function generally works in a loop, processing one file in each loop, while the processing logic is transparent to IOPA. The other two interface functions, i.e., *IOPA_Init()* and *IOPA_Close()*, are used to initialize and terminate IOPA, respectively.

**Table 1:** IOPA programming interface.

Interface function	Description
void IOPA_Init(IOPA_config* program_config)	Initialize IOPA. The user configuration is listed in Fig 3.
int IOPA_CreateThreads(void* (*function)(void*),void* args,int max_thread_number)	Create multiple working threads. The arguments include the user-defined thread function and its parameters and the expected maximum number of threads.
void IOPA_WaitComplete()	Wait for the termination of all working threads.
void IOPA_Close()	Destroy the data structures. Exit the program.

**Fig 3:**
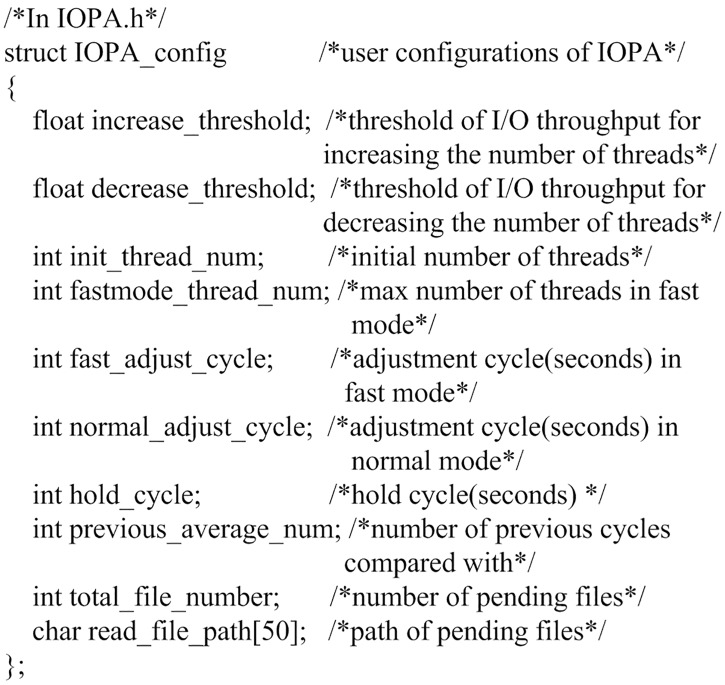
User configuration for IOPA.

**Fig 4:**
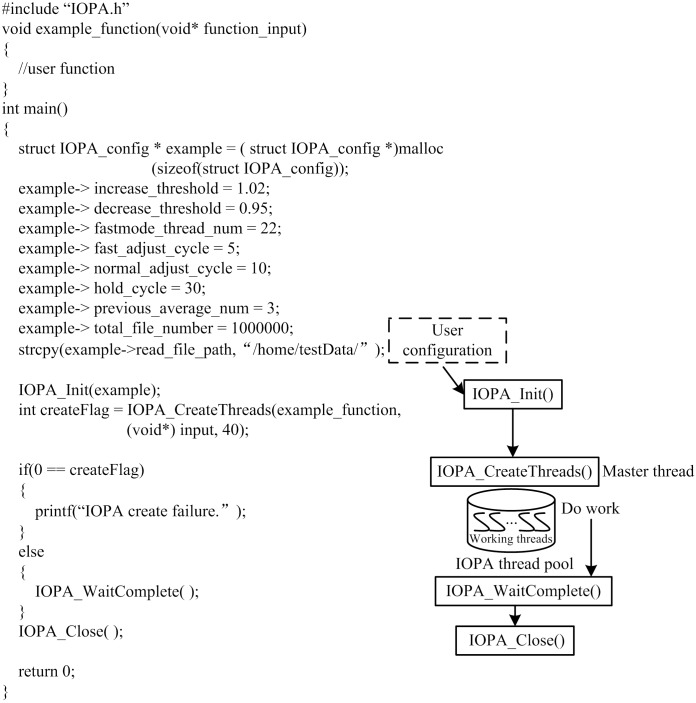
Example program and function call relationships of IOPA.

Fig 4 presents an example program to demonstrate the usage of the IOPA programming interface. The relationship between the programming interface and the application program is also shown in this figure.

## 4 System architecture and implementation

To obtain a degree of parallelism that matches the I/O capability of the system, IOPA periodically monitors the throughput of the I/O sub-system and uses the parallelism adjustment algorithm to calculate the appropriate number of threads; it then adjusts the number of threads when necessary by using the IOPA thread pool.

**Fig 5:**
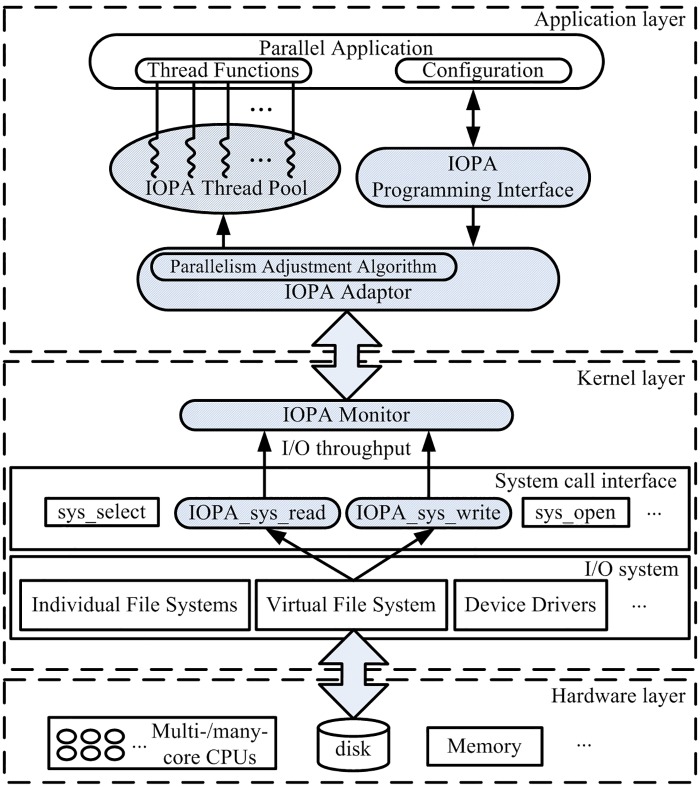
IOPA system architecture.

Fig 5 shows the IOPA system architecture. The overall system is composed of three layers: hardware layer, kernel layer and application layer. The shadowed parts in Fig 5 are IOPA modules.

In the kernel layer, IOPA uses *IOPA_sys_read* and *IOPA_sys_write* as probes to monitor file system throughput. Using program instrumentation, *IOPA_sys_read* and *IOPA_sys_write* collect the volume of data passing through *sys_read* and *sys_write* in the Linux kernel and then transmit the statistics to the *IOPA monitor*, which sums the statistics to obtain the I/O throughput of the system. The I/O throughput statistics are then transmitted to the *IOPA adaptor* at the application layer, which uses the parallelism adjustment algorithm to calculate the appropriate number of threads and to adjust the number of threads when necessary.

The user applications interact with *IOPA adaptor* via the *IOPA programming interface* introduced in the previous section; the working threads are maintained by the *IOPA adaptor* in a thread pool. Programmers need to provide the paths of pending files and the thread functions to IOPA.

**Fig 6:**
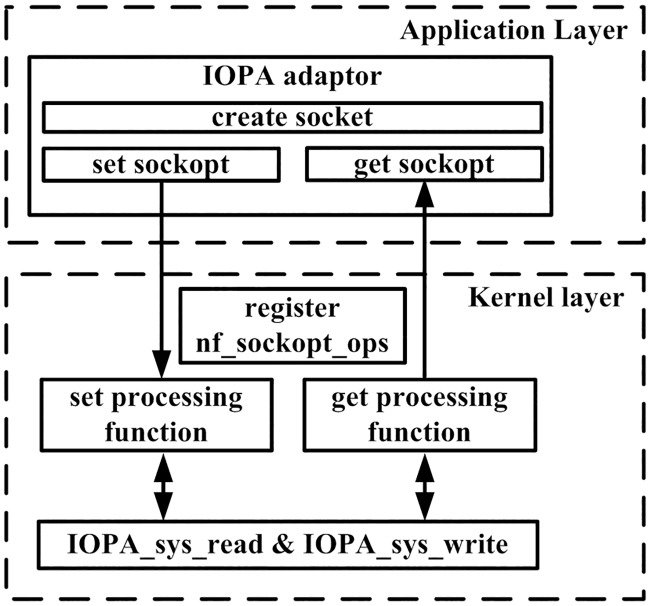
Detailed implementation of IOPA monitor.

Fig 6 shows the detailed implementation of *IOPA monitor*, in which *IOPA_sys_read* and *IOPA_sys_write* are implemented as LKM (Loadable Kernel Modules) [4], and the collected statistics are transmitted from the kernel to user-space via a socket [5].

## 5 Evaluation

### 5.1 Experimental setup

The system was evaluated in a server equipped with two six-core Intel Xeon 5650 processors and 12 GB of memory. By using hyper-threading support, the server can execute 24 threads simultaneously. To verify the applicability of IOPA, we, respectively, use a solid-state drive (Samsung 850 Pro, 256 G) and a hard disk drive (TOSHIBA enterprise hard-disk, 10000 RPM, 32 MB cache, 300 GB) in the evaluation. The operating system is Linux (Redhat 5.6 Enterprise Edition, 64-bit), with Linux kernel version 2.6.18-238.el5 and gcc 4.1.2.

The evaluation uses three sets of benchmark applications: 3 self-developed microbenchmark applications, Bzip2 [6] and 4 I/O-intensive applications from PARSEC 3.0 [7], comprising 8 programs in total, as listed in Table 2. The microbenchmarks are I/O-intensive applications. Bzip2 is also a typical I/O-intensive application. Although PARSEC 3.0 contains 13 programs, most are not I/O intensive; therefore, they are unsuitable for IOPA. Consequently, we selected only 4 I/O-intensive applications from them. The source codes of these benchmark applications can be accessed via the link, https://github.com/thomasball/IOPA.

**Table 2:** Benchmark applications.

Benchmark application		Dataset	Description
Microbenchmarks		The size of each kind of file (10 KB, 50 KB, and 100 KB) is floated on ±20%. The numbers of files of each size used are 2,000,000 (SSD) and 1,000,000 (HDD).	A series of simulated programs (reading files and then processing; the processing time is 10 ms). The test files used involved three file sizes: 10 KB, 50 KB and 100 KB, respectively. The outputs of this application are execution times.
Bzip2		The data comes from Corel-1k [8], which contains 1,000 images (256 × 384 pixels; the average size of each image is 30 KB), and we duplicate the images repeatedly to obtain enough data. The numbers of files used are 1,000,000 (SSD) and 500,000 (HDD).	A freely available, patent-free, high-quality data compressor [6] and an I/O intensive application. The outputs of this application are files with a.bz2 suffix, whose sizes are slightly smaller than those of the input files.
PARSEC3.0	Fluidanimate	The data comes from its own input_test dataset. We duplicate the files repeatedly to obtain enough data. The average size of each file is 160 KB. The numbers of files used are 200,000 (SSD) and 100,000 (HDD).	Fluidanimate uses an extension of the Smoothed Particle Hydrodynamics (SPH) method to simulate an incompressible fluid for interactive animation purposes [9]. The outputs of this application are files with an out suffix, whose sizes are similar to those of the input files.
	Blackscholes	The input data is generated by its own data generator. The average size of each file is 30 KB. The numbers of files used are 3,000,000 (SSD) and 1,500,000 (HDD).	Blackscholes calculates the prices for a portfolio of European options analytically with the Black-Scholes partial differential equation (PDE) [9]. The outputs of this application are files with an out suffix, whose sizes are smaller than those of the input files.
	Canneal	The data comes from its own input_test dataset. We duplicate the files repeatedly to obtain enough. The average size of each file is 3.3 MB. The numbers of files used are 10,000 (SSD) and 5,000 (HDD).	Canneal uses cache-aware simulated annealing (SA) to minimize the routing cost of a chip design [9]. The outputs of this application are computed values, not files.
	Bodytrack	The data comes from its own input_test dataset. We duplicate the folders repeatedly to obtain enough. The average size of each folder is 2.4 MB. The numbers of folders used numbers are 2,000 (SSD) and 1,000 (HDD).	The Bodytrack computer vision application is an Intel RMS workloadthat tracks the 3D pose of a markerless human body with multiple cameras through an image sequence [9]. The outputs of this application are files with a bmp suffix, whose sizes are smaller than those of the input files.

Each benchmark application was first executed with 8, 16, 24 and 32 fixed threads, respectively, and then executed under IOPA. The I/O throughput was recorded during each execution. Considering that the execution times for different threads were diverse and long, the results of the first 600 seconds are shown in the experiment results.

To ensure accuracy, each application was executed 3 times, successively, and the average I/O throughput and execution time were recorded. Moreover, the file buffers of the operating system were cleared using the “*sync && echo 3 > /proc/sys/vm/drop_caches*” command prior to each execution.

**Fig 7:**
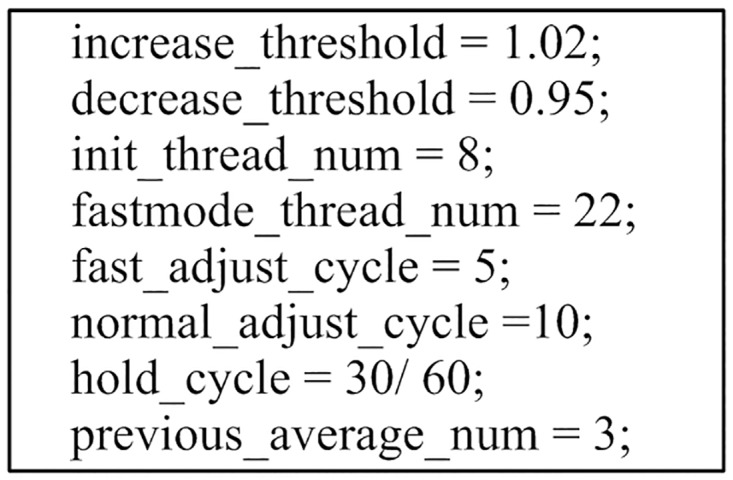
IOPA parameter values used in the experiments.

The IOPA parameter values used in the evaluation are shown in Fig 7, where *fastmode_thread_num* and *init_thread_num* are set to 90% and 30% of the number of logic cores, respectively.

### 5.2 Results

We used both a solid state and hard disk drive in the evaluation; the results are shown in sub-sections 5.2.1 and 5.2.2, respectively.

#### 5.2.1 Results with solid state drive

**Fig 8:**
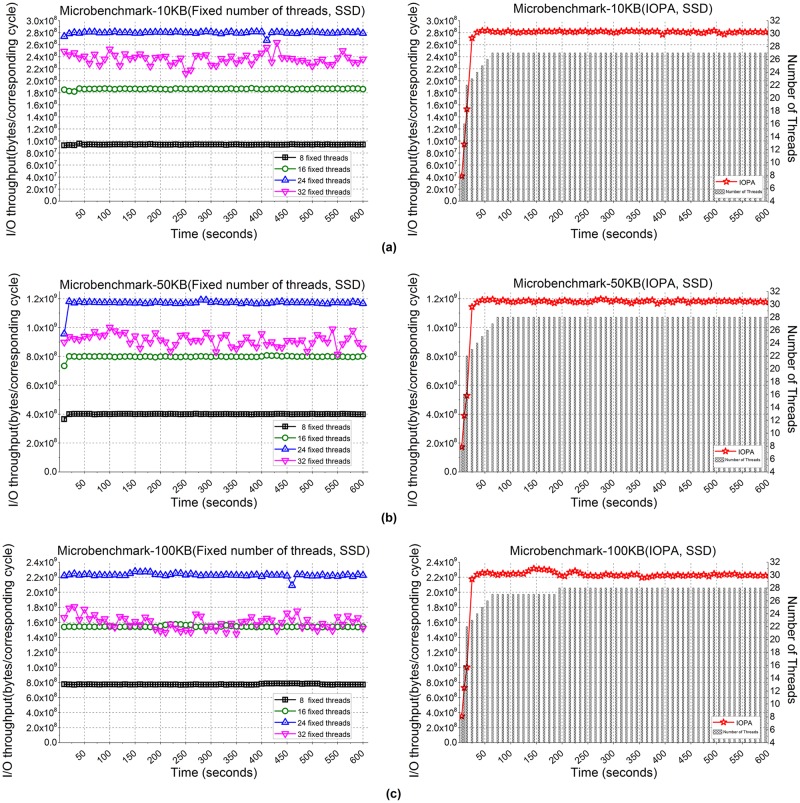
I/O throughput of microbenchmarks with an SSD. **(a)** File size: 10 KB files. **(b)** File size: 50 KB. (c) File size: 100 KB.

Fig 8 shows the performance of the microbenchmark applications when processing 2,000,000 files with size = 10 KB, 50 KB and 100 KB. Fig 8(a), 8(b) and 8(c) show the I/O throughput from using fixed numbers of threads and IOPA, respectively. As shown, the program with 24 fixed threads achieves the best performance among the programs using a fixed number of threads, while the performance of IOPA is close to and sometimes higher than the best value. Note that the optimal number of threads (24 in this test) is not only application-specific but also platform-dependent, while IOPA achieves the best or near-best performances adaptively.

Fig 8 also shows that under the control of IOPA, the number of threads increases rapidly (8→16→22) with a corresponding rapid increase in I/O throughput in *fast mode*. In this mode, the adjustment cycle is 5 seconds. After the number of threads reaches *fastmode_thread_num*(22), the parallelism adjustment algorithm of IOPA switches to *normal mode*, and the adjustment cycle changes to 10 seconds. When the number of threads reaches 26, the I/O throughput is relatively stable and IOPA maintains that the number of threads for a long time. The *hold_cycle* is 60 seconds. The *previous_average_num* is set to 3, which means that the algorithm compares the current I/O throughput with the average I/O throughput of the previous 3 cycles. The *hold_cycle* is 60 seconds.

**Fig 9:**
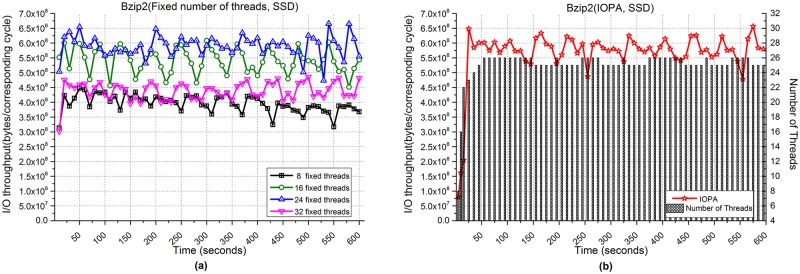
I/O throughput of Bzip2 with an SSD. **(a)** Fixed numbers of threads. **(b)** IOPA.

Fig 9 shows the performance of Bzip2 when processing 1,000,000 images. Fig 9(a) and 9(b) show the I/O throughput using fixed numbers of threads and IOPA, respectively. As shown in Fig 9(a), the program with 24 fixed threads achieves the best performance among the programs using a fixed number of threads, while the performance of IOPA in Fig 9(b) is close to and sometimes higher than the best value.

As shown in Fig 9(b), under IOPA, the number of threads increases rapidly (8→16→22) with a corresponding rapid increase in I/O throughput in *fast mode*. In this mode, the adjustment cycle is 5 seconds. After the number of threads reaches *fastmode_thread_num*(22), the parallelism adjustment algorithm of IOPA switches to *normal mode*, and the adjustment cycle changes to 10 seconds. Here, the *hold_cycle* is 30 seconds.

**Fig 10:**
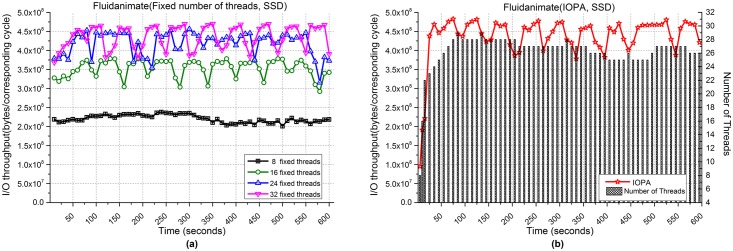
I/O throughput of Fluidanimate with an SSD. **(a)** Fixed numbers of threads. **(b)** IOPA.

Fig 10 shows the performance of Fluidanimate when processing 200,000files. Fig 10(a) and 10(b) show the respective I/O throughput when using fixed numbers of threads and IOPA. As shown in Fig 10(a), the program with 32 fixed threads achieves the best performance among the programs with a fixed number of threads. Meanwhile, the performance of IOPA shown in Fig 10(b) is close to and sometimes higher than the best value. The adjustment in *fast mode* is similar to that of bzip2. The *hold_cycle* is 30 seconds.

**Fig 11:**
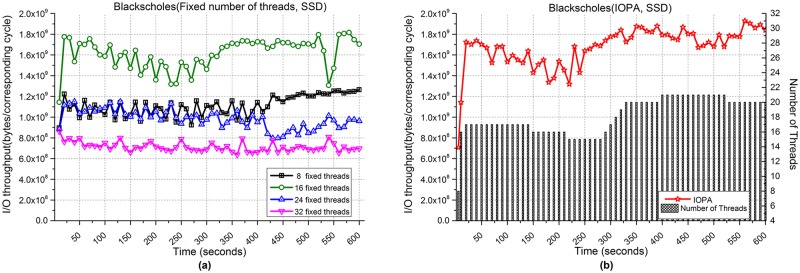
I/O throughput of Blackscholes with an SSD. **(a)** Fixed numbers of threads. **(b)** IOPA.

Fig 11 shows the performance of Blackscholes when processing 3,000,000 files. Fig 11(a) and 11(b) show the I/O throughput from fixed numbers of threads and IOPA, respectively. As shown in Fig 11(a), the program with 16 fixed threads achieves the best performance among the programs with a fixed number of threads.

Meanwhile, the performance of IOPA in Fig 11(b) is higher and more stable than the best value in Fig 11(a). Fig 11(b) also shows that under the control of IOPA, the number of threads increases rapidly (8→16) with a corresponding rapid increase of I/O throughput in *fast mode*. In this mode, the adjustment cycle is 5 seconds. After that, IOPA switches to *normal mode* and the adjustment cycle changes to 10 seconds. The *hold_cycle* is 60 seconds.

**Fig 12:**
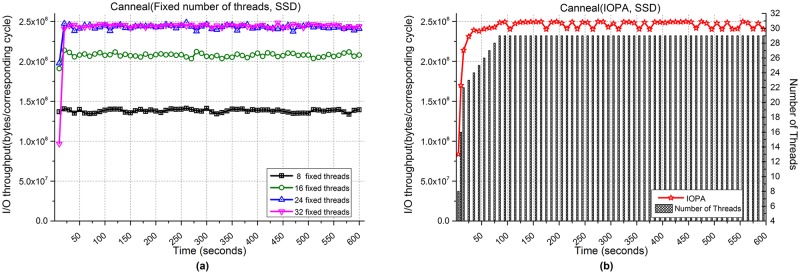
I/O throughput of Canneal with an SSD. **(a)** Fixed numbers of threads. **(b)** IOPA.

Fig 12 shows the performances of the Canneal application when processing 10,000 files. Fig 12(a) and 12(b) show the I/O throughput from using fixed numbers of threads and IOPA, respectively. As shown in Fig 12(a), the programs using 24 and 32 fixed threads achieve the best performances among the programs with a fixed number of threads, while the performance of IOPA in Fig 12(b) is close to and sometimes higher than the best value.

As shown in Fig 12(b), under the control of IOPA, the number of threads increases rapidly (8→16→22) and then the processing switches to *normal mode*. After 100 seconds, IOPA maintains the same number of threads for a long time. The *hold_cycle* is 60 seconds.

**Fig 13:**
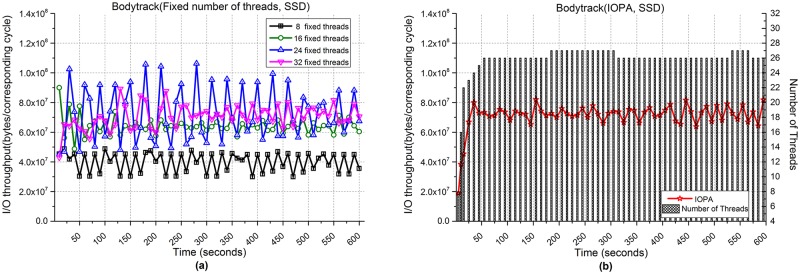
I/O throughput of Bodytrack with an SSD. **(a)** Fixed numbers of threads. **(b)** IOPA.

Fig 13 shows the performances of the Bodytrack application when processing 2,000 files. Fig 13(a) and 13(b) show the I/O throughput from fixed numbers of threads and IOPA, respectively. As shown in Fig 13(a), the program with 32 fixed threads shows the best performance among the programs with a fixed number of threads.

As shown in Fig 13(b), under the control of IOPA, the performance of IOPA in Fig 13(b) is close to and sometimes higher than the best value in Fig 13(a). The *hold_cycle* is 60 seconds.

Table 3 lists the execution time of the above benchmark applications for the SSD with fixed numbers of threads and IOPA, respectively. We can see that for most of the applications, IOPA achieves the highest efficiency. For Canneal, the program with 32 fixed threads achieves the highest efficiency, followed by IOPA. This is because the change in I/O throughput in Canneal is relatively constant and the adjustment of IOPA requires time. However, the gap in efficiency between IOPA and the best of the programs with a fixed number of threads is very small.

**Table 3:** Execution time of benchmark applications with SSD.

x	Thread number	Microbenchmark(10 KB)	Microbenchmark(50KB)	Microbenchmark(100 KB)	Bzip2	Fluidanimate	Blackscholes	Canneal	Bodytrack
Fixed thread-number	8	2615 s	2663 s	2695 s	1510 s	1574 s	1152 s	2522 s	4688 s
	16	1315 s	1334 s	1355 s	1105 s	983 s	792 s	1675 s	2871 s
	24	881 s	910 s	939 s	1015 s	827 s	1299 s	1485 s	2691 s
	32	1043 s	1165 s	1307 s	1351 s	798 s	1792 s	*1434 s*	2500 s
IOPA	Adaptive	*859 s*	*870 s*	*901 s*	*988 s*	*756 s*	*716 s*	1449 s	*2429 s*

#### 5.2.2 Results with hard disk drive

**Fig 14:**
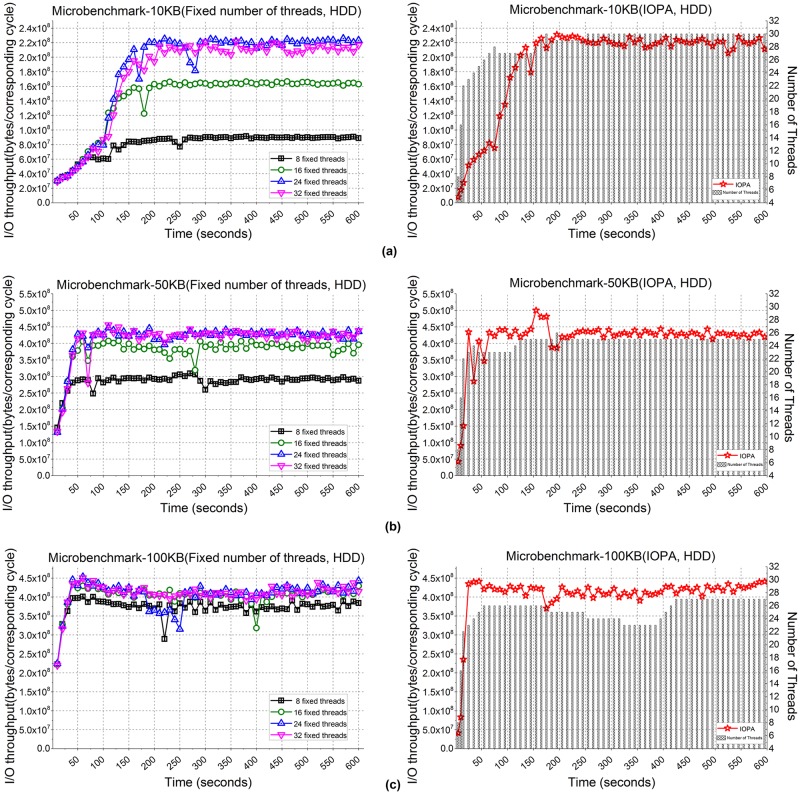
I/O throughput of microbenchmarks with an HDD. **(a)** File size: 10 KB files. **(b)** File size: 50 KB. **(c)** File size: 100 KB.

Fig 14 shows the performance of microbenchmark applications when processing 1,000,000 files with sizes of = 10 KB, 50 KB and 100 KB. The left and right part of Fig 14(a), 14(b) and 14(c) show the respective I/O throughput from using fixed numbers of threads and IOPA, where the corresponding cycle is 10 seconds. As shown in the figure, the program with 24 fixed threads achieves the best performance among the programs with a fixed number of threads, while the performance of IOPA is close to and sometimes higher than the best value, which is similar to the programs using the SSD.

Fig 14 also shows that under the control of IOPA, the number of threads increases rapidly (8→16→22) with a corresponding rapid in I/O throughput in *fast mode*. In this mode, the adjustment cycle is 5 seconds. After the number of threads reaches *fastmode_thread_num*(22), the parallelism adjustment algorithm of IOPA switches to *normal mode* and the adjustment cycle changes to 10 seconds. The *hold_cycle* is 60 seconds.

**Fig 15:**
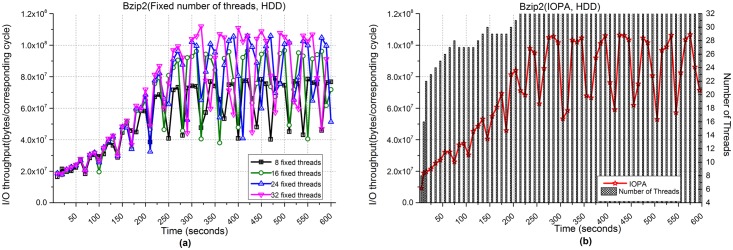
I/O throughput of Bzip2 with an HDD. **(a)** Fixed numbers of threads. **(b)** IOPA.

Fig 15 shows the performance of Bzip2 when processing 500,000 images. Fig 15(a) and 15(b) show the I/O throughput from using fixed numbers of threads and IOPA, respectively. Because the hardware structures of SSDs and HDDs are different and reading and writing of an HDD is subject to its head, fluctuations of I/O throughput are greater when employing HDDs than those when employing SSDs.

As shown in Fig 15(a), the program with 32 fixed threads achieves the best performance among the programs with a fixed number of threads, while the performance of IOPA in Fig 15(b) is close to and sometimes higher than the best value. The *hold_cycle* is 30 seconds.

**Fig 16:**
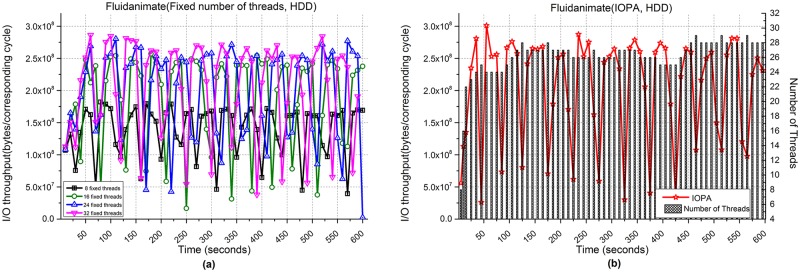
I/O throughput of Fluidanimate with an HDD. **(a)** Fixed numbers of threads. **(b)** IOPA.

Fig 16 shows the performance of Fluidanimate when processing 100,000 files. Fig 16(a) and 16(b) show the I/O throughput from using fixed numbers of threads and IOPA, respectively. As shown in Fig 16(a), the program with 32 fixed threads achieves the best performance among the programs with a fixed number of threads. Meanwhile, the performance of IOPA in Fig 16(b) is close to and sometimes higher than the best value. The *hold_cycle* is 30 seconds.

**Fig 17:**
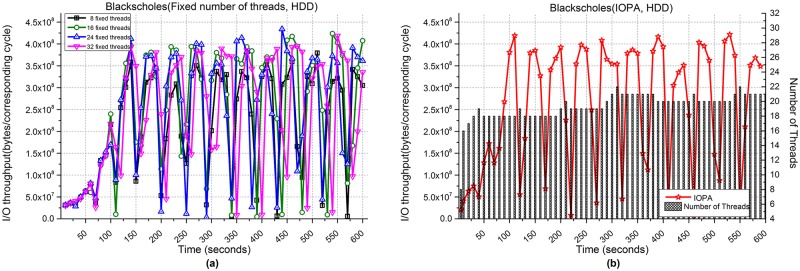
I/O throughput of Blackscholes with an HDD. **(a)** Fixed numbers of threads. **(b)** IOPA.

Fig 17 shows the performance of Blackscholes when processing 1,500,000 files. Fig 17(a) and 17(b) show the I/O throughput from using fixed numbers of threads and IOPA, respectively. As shown in Fig 17(a), the program with 16 fixed threads achieves the best performance among the programs with a fixed number of threads.

Fig 17(b) also shows that under the control of IOPA, the performance is higher and more stable than the best value in Fig 17(a). The *hold_cycle* is 60 seconds.

**Fig 18:**
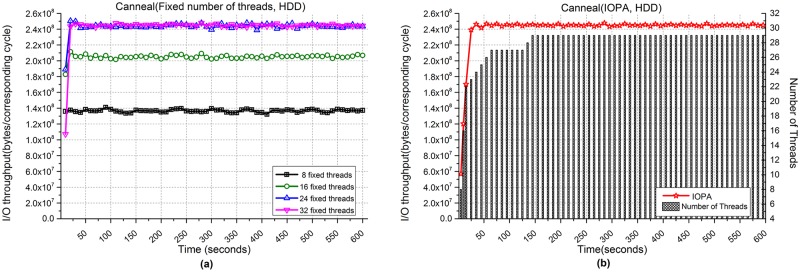
I/O throughput of Canneal with an HDD. **(a)** Fixed numbers of threads. **(b)** IOPA.

Fig 18 shows the performances of the Canneal application when processing 5,000 files. Fig 18(a) and 18(b) show the respective I/O throughput when using fixed numbers of threads and IOPA, respectively. As Canneal has few write operations, the fluctuation of I/O throughput is not significant. As shown in Fig 18(a), the programs with 24 and 32 fixed threads achieve the best performance among the programs with a fixed number of threads, while the performance of IOPA in Fig 18(b) is close to the best value. The *hold_cycle* is 60 seconds.

**Fig 19:**
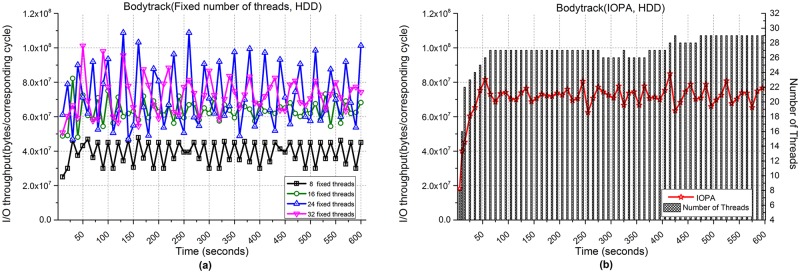
I/O throughput of Bodytrack with an HDD. **(a)** Fixed numbers of threads. **(b)** IOPA.

Fig 19 shows the performances of the Bodytrack application when processing 1,000 files. Fig 19(a) and 19(b) show the I/O throughput from using fixed numbers of threads and IOPA, respectively. As shown in Fig 19(a), the program with 32 fixed threads achieves the best performance among the programs with a fixed number of threads.

As shown in Fig 19(b), under the control of IOPA, the performance of IOPA in Fig 19(b) is close to and sometimes higher than the best value in Fig 19(a). The *hold_cycle* is 30 seconds.

Table 4 lists the execution time of the above benchmark applications for the HDD when using fixed numbers of threads and IOPA, respectively. Similar to the results for the SSD, for most of the applications, IOPA still achieves the highest efficiency. For Canneal, IOPA takes second place; the reason is similar to that for an SSD.

**Table 4:** Execution time of benchmark applications with HDD.

Programs	Thread-number	Microbenchmark(10 KB)	Microbenchmark(50 KB)	Microbenchmark(100KB)	Bzip2	Fluidanimate	Blackscholes	Canneal	Bodytrack
Fixed thread-number	8	1514 s	1859 s	2799 s	5496 s	1247 s	2913 s	1275 s	2344 s
	16	859 s	1393 s	2583 s	4733 s	924 s	2572 s	849 s	1439 s
	24	659 s	1272 s	2572 s	4471 s	880 s	2610 s	732 s	1250 s
	32	687 s	1279 s	2550 s	4296 s	849 s	2641 s	*715 s*	1253 s
IOPA	Adaptive	*604 s*	*1167 s*	*2309 s*	*4157 s*	*825 s*	*2380 s*	726 s	*1229 s*

As shown in Table 2, the number of processing files when using an SSD is double of that when using an HDD. We can see that in Tables 3 and 4, the execution time of most benchmark applications when using an SSD is shorter than that when using an HDD. The reason is that an SSD has a higher read/write speed than does an HDD. For the Canneal and Bodytrack benchmarks, the advantage of an SSD is not as obvious as for the other benchmarks. This is because Canneal and Bodytrack have relatively longer file processing times than do the other benchmark applications. Moreover, regardless of whether an SSD or an HDD is used, we can see that IOPA is more efficient than programs with a fixed numbers of threads for most of the benchmark applications. The performance of IOPA is very stable and IOPA has good applicability.

## 6 Related work

With the development of multi-/many-core processors, data intensive applications must increasingly be written as parallel programs. Much work has focused on parallelism adjustment. In a system equipped with multi-/many-core processors, I/O bandwidth can easily become a system bottleneck; thus, there has also been some work involving I/O aware to utilize I/O bandwidth more reasonably.

### 6.1 Parallelism adjustment

Arun Raman et al. [10] designed an API named DoPE (Degree of Parallelism Executive). Programmers can use DoPE to express parallelism options in nested loops just once; then DoPE dynamically optimizes the parallelism options at runtime. The work of [11] introduced a strategy named Few-to-Many (FM), which used incremental parallelism to reduce the high-percentile latency in interactive services. Based on service demand profiles and hardware parallelism, FM employs an offline phase to create a cycle Table, which forms the basis and provides guidance for adjusting the degree of parallelism. An evaluation illustrated that FM can reduce tail latency significantly. In [12], it was found that some multi-threaded applications scale well for small numbers of cores but poorly for large numbers of cores. This paper used hardware counters to discover that the applications are limited by memory-bandwidth. Energy can be saved by regulating the number of threads. The work of [13] targeted the Energy-delay Product (EDP) as the main optimization strategy. This paper used an extra helper-thread to assist multi-threaded programs in determining the appropriate numbers of CPUs and threads, and it can significantly reduce EDP. A model called ParallelismDial (PD) was proposed in [14]. PD can adapt a program's degree of parallelism continuously and dynamically by employing a holistic metric to optimize program execution. This model was evaluated with TBB [15] and Prometheus [16]. The result showed PD's high efficiency and ability to save energy. Similar to [14], [17] proposed a system named Varuna to model a program’s scalability and then dynamically and continuously adapt the program's parallelism. Furthermore, Varuna can optimize a program's execution in Min-time and Min-consumption and it is programming model independent. In [18], the Thread Tailor was proposed. Firstly, it conducts an offline analysis to estimate the type and communications of threads at runtime. Then, Thread Tailor combines the threads based on the collected offline information and the current hardware resources to suit the needs of the target system. This method can improve application performance. The work of [19] reported that [18] required offline information for guidance and cannot adapt a program's degree of parallelism dynamically at runtime. Therefore, they proposed a novel online model to adapt the program's degree of parallelism. Compared with previous models, this model is more efficient; a detailed evaluation was not included in this paper. The approach in [20] was named T-OPT, which determined the optimal number of threads by invoking an algorithm iteratively during multi-threaded program execution; the adjustment is limited in a given program region.

### 6.2 I/O aware

The work of [21] stated that SAT (Synchronization-Aware Threading) and BAT (Bandwidth-Aware Threading) can be the feedback-driven parameters to predict the optimal number of threads dynamically during runtime. Before program execution, these methods require a training phase to determine the initial number of threads. The Cluster R-aware Under-subscribed Scheduling of Threads (CRUST) was extended in [22] to propose an SMT-aware CRUST, which can obtain the behaviors of a program written in OpenMP[23] by leveraging hardware performance counter information to automatically determine the optimum thread count. An experiment on an Intel Xeon Phi [24] revealed ideal results. Yizhe Wang et al. [25] focused on the relationship between I/O requests and progress of thread execution and proposed a user-level scheme named iharmonizer for OpenMP programs. iharmonizer streamlines the I/O requests of multiple threads in OpenMP programs, and it is transparent to programmers. Xuechen Zhang et al. [26] met the QoS requirements for I/O-intensive programs and proposed a scheme named U-Shape to support end-users' QoS goals. U-Shape provides an API that programs can use to obtain the corresponding I/O requests. By employing machine learning to analyze the information and schedule the I/O requests, U-Shape guarantees the programs' QoS requirements.

Compared with the related work, this paper focuses on controlling the degree of parallelism in I/O-intensive applications. By adjusting the degree of parallelism in programs automatically based on I/O throughput and the available computing resources, this approach achieves a tradeoff between parallelism and I/O throughput, thus improving the overall performance of the system.

## 7 Conclusion

Parallelism adjustment is both a critical optimization in parallel applications and an academic research hotspot. Due to the performance gap between a CPU and an I/O sub-system, I/O bandwidth can easily become a system bottleneck; therefore, it is necessary to balance the computing resources and the I/O bandwidth by adjusting the number of I/O threads. This paper proposes IOPA, an I/O parallelism control mechanism that can adapt to the I/O needs and capabilities of a system. The IOPA monitors the load of an I/O sub-system and correspondingly adjusts the number of I/O threads in applications using a parallelism adjustment algorithm. A programming interface is also provided to programmers. Evaluations using both an SSD and an HDD prove that IOPA can adjust the number of threads in a timely manner based on the real-time I/O bandwidth and make parallel applications to achieve higher efficiency than programs that use a fixed number of threads.
